# Correction: Unveiling the role of cobalt in the product regulation for CO_2_ hydrogenation to light olefins over alumina-supported Co–Fe catalysts

**DOI:** 10.1039/d5sc90185e

**Published:** 2025-08-22

**Authors:** Zhihao Liu, Wenlong Song, Peipei Zhang, Jiaming Liang, Chengwei Wang, Chufeng Liu, Hanyao Song, Baojian Chen, Kangzhou Wang, Guangbo Liu, Xiaoyu Guo, Yingluo He, Xinhua Gao, Jianli Zhang, Guohui Yang, Noritatsu Tsubaki

**Affiliations:** a Department of Applied Chemistry, School of Engineering, University of Toyama Gofuku 3190 Toyama 930-8555 Japan; b State Key Laboratory of High-efficiency Coal Utilization and Green Chemical Engineering, College of Chemistry and Chemical Engineering, Ningxia University Yinchuan 750021 Ningxia China; c CNOOC Institute of Chemical & Advanced Materials Beijing 102209 China; d School of Materials and New Energy, Ningxia University Yinchuan 750021 Ningxia China; e Key Laboratory of Biofuels, Qingdao Institute of Bioenergy and Bioprocess Technology, Chinese Academy of Sciences Qingdao 266101 China

## Abstract

Correction for ‘Unveiling the role of cobalt in the product regulation for CO_2_ hydrogenation to light olefins over alumina-supported Co–Fe catalysts’ by Zhihao Liu *et al.*, *Chem. Sci.*, 2025, **16**, 14140–14151, https://doi.org/10.1039/D5SC04407C.

It has come to the authors’ attention that panel (a) in Fig. 6 was inadvertently duplicated in place of panel (b), resulting in both panels showing the same image. The corrected Fig. 6 is shown below. This correction does not affect the results and conclusions of the study.

**Fig. 1:**
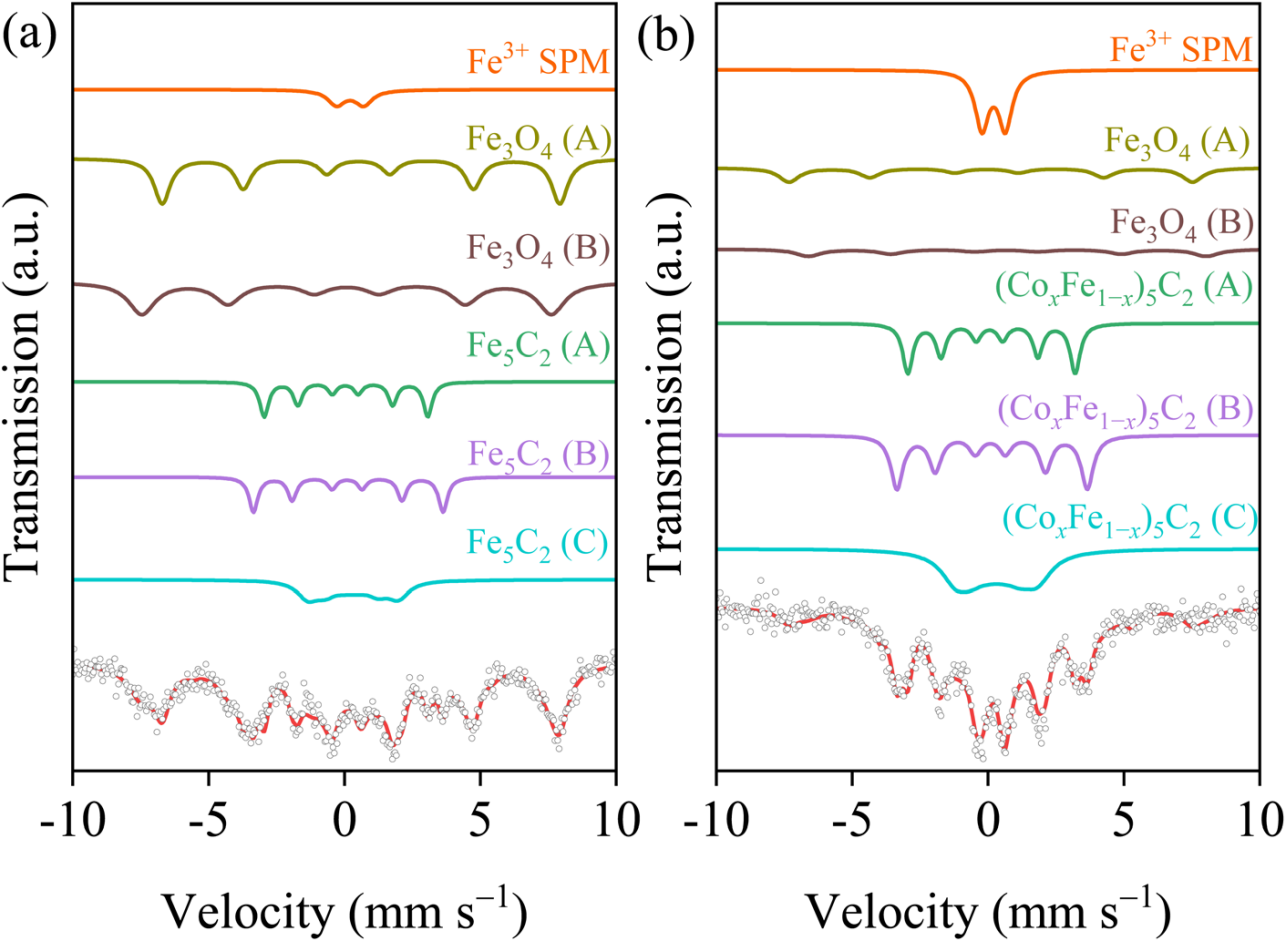
^57^Fe Mössbauer spectra of the spent (a) Co0Fe and (b) Co1Fe2 catalysts.

The Royal Society of Chemistry apologises for these errors and any consequent inconvenience to authors and readers.

